# Ocular Complications of Diabetes and Therapeutic Approaches

**DOI:** 10.3390/jcm11175170

**Published:** 2022-09-01

**Authors:** Pedro Romero-Aroca

**Affiliations:** Ophthalmology Service, University Hospital Sant Joan, Institut de Investigacio Sanitaria Pere Virgili (IISPV), Universitat Rovira & Virgili, 43002 Tarragona, Spain; romeropere@gmail.com

Diabetes mellitus, more simply called diabetes, is a chronic condition that occurs when blood glucose levels rise because the body cannot produce any or enough of the hormone insulin or cannot effectively use the insulin it produces [1].

Diabetes is a major driver of mortality worldwide, although its impact varies by region. Thus, excluding the mortality risks associated with the COVID-19 pandemic, approximately 6.7 million adults aged between 20 and 79 died because of diabetes or its complications in 2021.

An estimated 537 million adults aged 20–79 years are currently living with diabetes in the world: this represents 10.5% of the world’s population in this age group. The total number is predicted to rise to 643 million (11.3%) by 2030 and to 783 million (12.2%) by 2045 [2].

Furthermore, 240 million people are living with undiagnosed diabetes worldwide, meaning almost one-in-two adults with diabetes are unaware they have the condition.

There are two major types of diabetes mellitus (DM): type 1, caused by an autoimmune process in which the body’s immune system attacks the insulin-producing beta cells of the pancreas, and type 2, in which hyperglycemia is the result of the inability of the body’s cells to respond fully to insulin, a condition termed insulin resistance. With the onset of insulin resistance, the hormone is less effective and, in due course, prompts an increase in insulin production. Over time, inadequate production of insulin can develop as a result of failure of the pancreatic beta cells to keep up with demand. Type 2 DM is the most common type of diabetes, accounting for over 90% of all diabetes worldwide [3].

The morbidity and mortality of diabetes mellitus is given by the different complications at the systemic level. The majority of such complications are secondary to the involvement of large vessels or macroangiopathy (lower-limb ulcers, heart disease or stroke) or to the involvement of small vessels or microangiopathy, such as neurovascular involvement of the retina, nephropathy and peripheral sensory neuropathy.

At the ocular level, diabetic retinopathy (DR) is the best known for doctors and patients, being the fifth cause of low vision and blindness in young adults in the world. An interesting fact is that although the age-standardized prevalence of blindness worldwide for all modeled causes consistently showed a decrease between 1990 and 2020, there was a notable exception in the case of diabetic retinopathy, for which prevalence increased in the world [4].

DR is often not diagnosed until the first symptoms of vision loss appear. In DM patients, poorly controlled blood glucose levels have a great impact on the risk of DR. Type 1 DM patients spend many more years of their lives in this situation than type 2 DM, and therefore, it is a group to be especially considered. However, DR impacts type 2 patients much more, as they have often been underdiagnosed. With all this, the conclusion is that the greatest burden for the detection of DR is associated with type 2 DM [1].

DR has traditionally been considered a microvascular disease of the retina; however, mounting evidence suggests that retinal neurodegeneration is an early event in the pathogenesis of DR that could contribute to the development of microvascular abnormalities [5,6].

Regarding detection, the screening of diabetic patients to detect early forms is a global need [7]. DR screening is different from population screening programs because it focuses on people who are already known to have a condition. Checking the eyes of a person with diabetes and providing appropriate treatment is an evidence-based intervention that reduces the risk of visual impairment and blindness and should be part of routine care for people with DM. Currently, screening aids are beginning to be introduced in the form of the application of artificial intelligence through image analysis, in addition to the introduction of algorithms for detecting the risk of developing DR: techniques that will allow personalized control of DM patients.

The treatment of DR according to the Diabetic Retinopathy Preferred Practice Pattern^®^ of the American Academy of Ophthalmology [8] includes following a healthy diet and lifestyle, medical management, timely ophthalmologic evaluation and treatment under the care of an ophthalmologist. Treatment with laser, anti-VEGF agents or intravitreal corticosteroids is cost-effective in controlling DR to varying degrees [9], and intravitreal anti-vascular endothelial growth factor (anti-VEGF) agents are effective in treating DME with central involvement and vision loss. Laser photocoagulation surgery is recommended for DME not affected by the center, and panretinal photocoagulation surgery remains the main treatment for PDR. Analyses of two clinical trials showed that treatment for DR may be 90% effective in preventing severe vision loss (visual acuity <5/200) using current therapeutic treatment strategies [10].

Although DR is the best-known form of ocular involvement due to diabetes, there are other conditions that are no less frequent, such as lens involvement, neovascular glaucoma, non-arteritic ischemic optic neuropathy or conditions of the nerves responsible for extrinsic ocular motility [11].

Although cataracts due to direct hyperglycemia were more frequent years ago and can still be found in undeveloped countries, in the Western world, its appearance is infrequent, although it is not exceptional, and cataracts may appear in young patients with type 1 diabetes. Another way of affecting the lens is the acceleration of a pre-existing cataract, which, due to the association of diabetes, can cloud the lens earlier, requiring surgery earlier than if there is no cataract.

Another form of ocular involvement is neovascular glaucoma, secondary to the formation of new vessels at the angle of the chamber (between the root of the iris and the cornea) due to the formation of large areas of ischemia in the peripheral retina in patients with severe diabetic or proliferative retinopathy.

Non-arteritic ischemic optic neuropathy secondary to poor diabetes control and its association with high blood pressure, which produces ischemia at the papilla level with the association of loss of visual acuity and an altitude defect in the visual field that recovers with difficulty, results in vision defects in a large number of cases.

Finally, diplopia may appear due to paralysis and paresis of the extrinsic ocular muscles secondary to poor glycemic control and normally associated with poor blood pressure control. These cases are usually transient and recover within six months if blood pressure control is restored. An important fact is that paralysis and paresis are secondary to diabetes mellitus with respect pupillary motility; that is, pupillary reflexes are normal in these patients.

In conclusion, the knowledge of ocular DM complications, its screening and treatment with the application of modern technologies, including artificial intelligence, and the continuous search for and development of new techniques are crucial to making ocular complications of DM treatment safer and more efficient.

As the Guest Editors, we would like to sincerely thank the reviewers for their insightful remarks and the *JCM* team’s support. Ultimately, we heartily thank all of the contributing authors for their valuable input.
